# Brief Report: The Impact of Disease Stage on Early Gaps in ART in the “Treatment for All” Era—A Multisite Cohort Study

**DOI:** 10.1097/QAI.0000000000002605

**Published:** 2020-12-17

**Authors:** Ingrid T. Katz, Nicholas Musinguzi, Kathleen Bell, Anna Cross, Mwebesa B. Bwana, Gideon Amanyire, Stephen Asiimwe, Catherine Orrell, David R. Bangsberg, Jessica E. Haberer

**Affiliations:** a Department of Medicine, Brigham and Women's Hospital, Boston, MA;; b Harvard Medical School, Boston, MA;; c Harvard Global Health Institute, Cambridge, MA;; d Mbarara University of Science and Technology, Mbarara, Uganda;; e Massachusetts General Hospital Center for Global Health, Boston, MA;; f Desmond Tutu HIV Foundation, University of Cape Town Medical School, Cape Town, South Africa;; g Kabwohe Clinical Research Center, Kabwohe, Uganda; and; h Oregon Health and Science University-Portland State University School of Public Health, Portland, OR.

**Keywords:** HIV, early gaps, South Africa, Uganda

## Abstract

**Background:**

Adoption of “Treat All” policies has increased antiretroviral therapy (ART) initiation in sub-Saharan Africa; however, unexplained early losses continue to occur. More information is needed to understand why treatment discontinuation continues at this vulnerable stage in care.

**Methods:**

The Monitoring Early Treatment Adherence Study involved a prospective observational cohort of individuals initiating ART at early-stage versus late-stage disease in South Africa and Uganda. Surveys and HIV-1 RNA levels were performed at baseline, 6, and 12 months, with adherence monitored electronically. This analysis included nonpregnant participants in the first 6 months of follow-up; demographic and clinical factors were compared across groups with χ^2^, univariable, and multivariable models.

**Results:**

Of 669 eligible participants, 91 (14%) showed early gaps of ≥30 days in ART use (22% in South Africa and 6% in Uganda) with the median time to gap of 77 days (interquartile range: 43–101) and 87 days (74, 105), respectively. Although 71 (78%) ultimately resumed care, having an early gap was still significantly associated with detectable viremia at 6 months (*P* ≤ 0.01). Multivariable modeling, restricted to South Africa, found secondary education and higher physical health score protected against early gaps [adjusted odds ratio (aOR) 0.4, 95% confidence interval (CI): 0.2 to 0.8 and (aOR 0.93, 95% CI: 0.9 to 1.0), respectively]. Participants reporting clinics as “too far” had double the odds of early gaps (aOR 2.2: 95% CI: 1.2 to 4.1).

**Discussion:**

Early gaps in ART persist, resulting in higher odds of detectable viremia, particularly in South Africa. Interventions targeting health management and access to care are critical to reducing early gaps.

## BACKGROUND

The widespread availability of antiretroviral therapy (ART) throughout sub-Saharan Africa has transformed the HIV epidemic across the region, increasing the number of people on treatment from 100,000 in 2004 to 15.4 million in 2017.^1^ This increase in availability has dramatically impacted cumulative ART initiation, with some regions experiencing up to a 17.6 percentage point increase from 6–18 months pre-expansion to 6–18 months postexpansion.^2^ After the adoption of national treat-all policies in 6 sub-Saharan African nations, statistically significant increases in rapid ART initiation were observed in 4 countries, with sustained or amplified improvements in adherence.^3^

In South Africa, 70.6% of the 7.9 million people living with HIV (PLWH) ages 15–64 are currently on treatment, with 87.5% of those on treatment virally suppressed.^4^ This represents a 2-fold increase in the past decade,^5^ accelerated by the expansion in ART eligibility to Treat All as of September 2016.^6^ In Uganda, where the treatment guidelines were expanded to Treat All as of November 2016,^3^ treatment initiation numbers are similar with for PLWH ages 15–64 with estimates of 89.3% of PLWH on ART and of which 90.6% are virally suppressed.^7^

Although there are data to suggest the impact of guideline expansions has increased early ART initiation in sub-Saharan Africa,^2,3^ early gaps in treatment (ie, discontinuation of ≥30 days within the first 6 months) persist.^6^ The first 6 months of treatment after initiation are crucial to long-term adherence and immunological and virologic success, with 6-month CD4 count and viral load being the 2 most important factors in prediction of progression to AIDS or death.^8^ Because of the clinical significance of this period, more information is needed to understand why treatment discontinuation continues at this vulnerable stage in care.

We leveraged a prospective observational cohort study of individuals initiating ART at early-stage versus late-stage disease to understand how disease stage impacts early gaps in treatment in 2 countries in the region—South Africa and Uganda. The data collected during this observational cohort study allowed for an in-depth investigation into sociobehavioral factors that could affect health decision-making, care access, and treatment-related behavior beyond the health status. We identified clinical and psychosocial predictors of gaps in these 2 groups and hypothesized that individuals starting ART with late-stage disease have faced long-term marginalization and challenges in coping with their HIV diagnosis and, therefore, would be more likely to halt treatment. We based this hypothesis on the literature that suggests that patients who present late to care are less likely to remain in care.^9,10^

## METHODS

### Study Design and Setting

This analysis used data from the Monitoring Early Treatment Adherence (META) Study (NCT02419066), a prospective, observational study designed to assess ART adherence among 2 cohorts of men and women initiating ART in routine care in Cape Town, South Africa, and southwestern Uganda. Full protocol details have been published previously.^11^ Our previous study found that one cohort initiated treatment with early-stage HIV infection (CD4 > 350 cells/mL) and one initiated with late-stage HIV infection (CD4 < 200 cells/mL). Participants were seen at 0, 6, and 12 months for administration of sociobehavioral questionnaires and HIV-1 RNA levels. Adherence was monitored electronically in real-time (Wisepill wireless adherence device; Wisepill Technologies, South Africa). The parent study showed that adherence data varied by site. Given this, we chose to analyze data from each site separately. In Uganda, adherence over 12 months was not significantly different between individuals with early-stage and late-stage initiation, with an overall median of 89%. In South Africa, median adherence rates over 12 months were 77% and 52%, respectively.^11^

### Analysis

For this analysis, early gaps in ART were defined as ≥30 consecutive days without evidence of adherence in the first 6 months of treatment among all individuals initiating ART. Our previous study looked at ≥7 days as a measure of viremia. We chose to look at ≥30 consecutive days as a measure of lack of care engagement which is comparable with previous studies.^12–15^ Demographic and clinical factors at baseline (chosen after being identified in the previous literature as potential factors of treatment initiation^16^ and adherence^17^) were compared across groups using χ^2^ for categorical and Wilcoxon rank sum test for continuous factors to identify potentially confounding covariates. Pregnant women were not included in this analysis because factors influencing care often differ substantially from nonpregnant individuals.^18^ Logistic regression models were used to estimate predictors of early gaps in ART usage.

In determining what variables to retain for the multivariable model, for each potential confounder, we fit a univariable model including the confounder alongside the study cohort and retained in the multivariable models all potential confounders with a significance level of *P* < 0.10. To distinguish nonuse of the adherence monitor versus true nonadherence during the recorded gap, we assessed the relationship between gaps and the 6-month viremia using χ^2^ tests. The Uganda and South Africa sites were analyzed separately because of numerous demographic and socioeconomic differences between them. Adjusted analyses were restricted to South Africa, given the limited numbers of participants with early gaps in Uganda.

### Ethical Considerations

Study procedures were approved by Ethical and Regulatory Committees at Partners Healthcare (Protocol P2014P002620), the Mbarara University of Science and Technology (Protocol 11/04–14), Uganda National Council for Science and Technology (Protocol HS 1667), University of Cape Town (Protocol 797/2014), and Western Cape province (Protocol WC2014_RP16_343) in South Africa. All participants provided written informed consent.

## RESULTS

### Participant Characteristics

Of the 904 people living with HIV enrolled in the META study between March 2015 and September 2016, 421 were in South Africa, and 483 were in Uganda. In total, adherence data were available at 6 months for 96% (n = 868); 77% (n = 669) were found eligible for this analysis, with 199 participants excluded because of pregnancy. The median age of analyzed participants was 33 years; approximately, 60% were female, and the study site was divided approximately 48%–52% between South Africa and Uganda (Table 1).

**TABLE 1.** Participant Demographics and Outcomes by the Site

Variable	Uganda N (%) or Median (IQR) N = 347	South Africa N (%) or Median (IQR) N = 322	*P*
Sociodemographic characteristics (baseline)			
Cohort			0.19
Early-stage disease	175 (50)	146 (45)	
Late-stage disease	172 (50)	176 (55)	
Female	198 (57)	204 (63)	0.10
Age	31 (26–39)	35 (29–42)	<0.001
Married	159 (46)	63 (20)	<0.001
Sexually active in the past 6 mo	267 (77)	280 (88)	0.001
Completed high-school education	193 (56)	274 (85)	<0.001
Literate in English or local language	295 (85)	303 (96)	<0.001
Employed	308 (89)	148 (46)	<0.001
Forced sex	29 (8)	13 (4)	0.02
Annual income (USD)	333 (74–740)	728 (0–2821)	<0.001
Structural barrier score	0 (0–3)	12 (8–18)	<0.001
Clinic “too far”	56 (16)	100 (31)	<0.001
Severe food insecurity	110 (32)	217 (67)	<0.001
Social support score (instrumental)	31 (23–40)	29 (21–41)	0.58
Social support score (emotional)	36 (25–44)	30 (21–43)	0.013
Stigma (perceived negative attitudes)	1 (0–3)	3 (1–4)	<0.001
Stigma (disclosure concerns)	3 (1–5)	3 (1–6)	0.78
Disclosed to anybody	289 (83)	266 (83)	0.75
Coping score	2.3 (2.0–2.4)	2.3 (2.3–2.6)	<0.001
Medical mistrust score	2 (1–2.5)	2 (2–2.5)	<0.001
Satisfaction score	3.5 (3.2–4)	3.0 (2.8–3.3)	<0.001
Physical health score	39 (36–43)	41 (36–47)	<0.001
Mental health score	47 (36–60)	37 (32–46)	<0.001
Probable depression	88 (25)	158 (49)	<0.001
Heavy alcohol usage	33 (10)	96 (30)	<0.001
First HIV test ≥30 days prior to enrolment	177 (51)	175 (60)	0.021
Other medications beside ART	286 (82)	75 (23)	<0.001
Outcomes			
Presence of 30-day interruptions	21 (6)	70 (22)	<0.001
Time (days) to interruption	87 (74–105)	77 (43–101)	0.14
6-month viral suppression	307 (89)	256 (80)	0.002

### Early Gaps in Treatment

Ninety-one individuals (14%) showed early gaps in ART use, 70 individuals from South Africa and 21 individuals from Uganda, with a median time from ART initiation to treatment gap of 77 days [interquartile range (IQR): 43–101] and 87 days (IQR: 74–105), respectively. The median duration of adherence gaps was 48 days (IQR: 36–56) among Ugandan participants and 45 days (IQR: 38–65) among South African participants. The proportion of early-ART and late-ART initiators with treatment gaps was 9.7% and 17.2%, respectively (*P* = 0.004). Although 71 (78%) of those with early gaps across sites ultimately resumed care, they were still more likely to have detectable viremia at month 6 (*P* ≤ 0.01). Among people who did not have a gap in treatment, 87.5% were virally suppressed versus 66.0% among those who had a gap in care (*P* < 0.001). Almost all participant sociodemographic characteristics differed across the 2 sites with a few exceptions (Table 1). In general, South African participants were older, were more likely to be depressed, more likely to be heavy alcohol consumers, and had a higher physical but lower mental health score.

Multivariable regression modeling was restricted to South Africa, given the limited numbers of early gaps among participants in Uganda. The multivariable model results are shown in Table 2. In the adjusted model, we found that secondary education and higher physical health score provided a protective effect against early gaps [(aOR 0.4, 95% CI: 0.2 to 0.8) and (aOR 0.93, 95% CI: 0.9 to 1.0), respectively]. Participants reporting the clinic to be too far had double the odds of early gaps (aOR 2.2: 95% CI: 1.2 to 4.1). There was no significant difference across early-stage versus late-stage disease, age, gender, marital status, or employment, despite the significant difference in overall adherence.

**TABLE 2.** Adjusted Analyses of Factors Associated With Early Gaps in ART Among South African Participants Living With HIV

Variable	Univariable Model			Multivariable Model		
	aOR	95% CI	*P*	aOR	95% CI	*P*
Cohort						
Late-stage vs. early-stage disease	1.80	1.03 to 3.13	0.037	1.46	0.78 to 2.73	0.243
Age (5 year effect)	1.06	0.93 to 1.20	0.391			
Female	1.18	0.67 to 2.09	0.560			
Sexually active in the past 6 mo	0.82	0.38 to 1.76	0.605			
Completed high-school education						
Completed vs. never completed high school	3.00	0.16 to 0.57	<0.001	0.33	0.16 to 0.67	0.002
Literate in English or local language	0.24	0.08 to 0.69	0.008			0.323
Employed	0.77	0.45 to 1.32	0.336			
Forced sex	0.71	0.15 to 3.31	0.660			
Annual income (per 100 USD)	0.99	0.98 to 1.01	0.318			
Structural barrier score	1.03	0.99 to 1.07	0.200			
Married or living together	0.55	0.25 to 1.18	0.124	0.43	0.18 to 1.01	0.053
Clinic “too far”	1.93	1.12 to 3.35	0.019	2.28	1.25 to 4.15	0.009
Physical health score	0.93	0.89 to 0.97	<0.001	0.93	0.89 to 0.97	0.001
Mental health score	1.00	0.97 to 1.03	0.996			
Social support score (instrumental)	0.99	0.96 to 1.02	0.464			
Social support score (emotional)	0.99	0.97 to 1.02	0.581			
Stigma (perceived negative attitudes)	1.04	0.89 to 1.20	0.630			
Stigma (disclosure concerns)	0.99	0.88 to 1.10	0.818			
Disclosed to anybody	0.94	0.46 to 1.92	0.864			
Coping score	1.15	0.73 to 1.82	0.554			
Medical mistrust score	1.02	0.62 to 1.67	0.948			
Satisfaction score	1.22	0.69 to 2.18	0.491			
Probable depression	1.36	0.79 to 2.32	0.270			
Heavy alcohol usage	0.83	0.46 to 1.51	0.550			
Other medications beside ART	1.47	0.80 to 2.68	0.21			

## DISCUSSION

Despite many successes in the global efforts to promote early and enduring treatment, early gaps in ART persist, resulting in higher odds of detectable viremia. These gaps remain significant for key vulnerable populations, particularly in South Africa, where we found 22% (70 of 322 participants) had an early gap in care, compared with 6% in Uganda. Although 71 (78%) of those with early gaps ultimately resumed care, having an early gap was still significantly associated with detectable viremia at 6 months. This finding is consistent with previous studies showing individuals in South Africa seem to be at a particular risk of early losses in care, with rates of loss as high as 25% in the first 6 months on treatment,^6,19–21^ preventing people living with HIV from achieving the long-term benefits of treatment. In this study, early gaps did not significantly differ between disease stage, suggesting that perception of health may contribute more than actual disease severity.

We originally hypothesized that individuals starting ART with late-stage disease have faced long-term marginalization and challenges in coping with their HIV diagnosis and, therefore, would be more likely to halt treatment. This hypothesis was based on our prior research^22^ showing ART-related decision-making is best understood within the larger context of risk perception, which posits that people make decisions about risks based on affect, stigma, and/or fear, and are highly loss averse.^23^ Although we did not find that disclosure/stigma were significantly associated with early losses, our findings may reflect a more nuanced interpretation of stigma. Our recent findings in another study in South Africa shows that internalized stigma may naturally decrease over time after an HIV diagnosis, whether someone is on treatment or not, because there is time to adjust to a diagnosis and acquire social support.^24^ Although we were unable to fully explore the impact of actual time to diagnosis on decision-making, this is an area that would benefit from future research.

In addition, those at a highest risk of early gaps in ART in South Africa appeared to be PLWH who reported that the clinic was “too far.” This finding is consistent with prior research in South Africa showing people living with HIV who struggle to remain in care often describe clinics as being hard to access and additionally may view health centers as stigmatizing and unwelcoming.^16,25,26^ Additional studies support our findings, citing poor testing accessibility, difficulties coordinating ART care, and pervasive stigma as contributing factors for risk of early gaps in ART.^27,28^

Ultimately, early losses impede the South African Government's ability to achieve population reductions in HIV by undermining the potential of the Prevention Access Campaign's Undetectable = Untransmittable (U = U) initiative.^29^ Beyond the implications for increased viral transmission, PLWH who drop out of care have high rates of mortality; 46% who drop out of public ART programs in sub-Saharan Africa ultimately succumb to the virus.^30^ The current national guidelines in South Africa require frequent client interaction with a health system that many find stigmatizing and located too far for easy access, with monthly clinical visits in the first 6 months after ART initiation. For those who drop out, the standard of care provides a clinic-initiated phone call and encouragement to re-establish care, but data from Khayelitsha township in the Western Cape show that this is insufficient for re-engagement.^31^

Given the multifactorial challenges that PLWH in South Africa face early in their care, differentiated service delivery models that use client-centered approaches to simplify and adapt services to reflect the preferences and needs of PLWH have the potential to improve retention in care for PLWH in South Africa.^32,33^ These differentiated care models are in line with the World Health Organization (WHO),^34^ the International AIDS Society guidelines,^35^ and the South African National Strategic Plan's consolidated guidelines^36^ and may remove some of the barriers identified in this study such as distance and education, by providing care within the community and supporting individuals with clinical navigation.^37,38^ Beyond providing flexible, client-centered care models, PLWH may benefit from interventions designed to promote resilience resources, focused on decreasing maladaptive coping strategies (eg, denial),^39^ promote new coping skills, improve social support, and reduce stigma for PLWH.^40^

Although this study provides valuable information about the sociobehavioral factors associated with early gaps in ART, it also presents a few key limitations. First, we recognize that the study was observational in nature, and therefore, the results of this study are limited to association, not causation. In addition, adherence monitoring may have influenced behavior. In addition, device nonuse may account for some nonadherence. As such, experiences may vary in the future and with long-term ART use. We had limited ability to comment on early gaps in Uganda, given the limited numbers of early gaps among individuals there, demonstrating that many PLWH in Uganda are successful in adhering to treatment early. We also did not measure drug resistance, which may have contributed to the presence of viremia among participants in this study.

In summary, this study allowed for in depth investigation of the sociobehavioral factors that contribute to early gaps in ART for PLWH in South Africa and Uganda. We found that the study population in South Africa had a higher rate of early gaps in ART compared with the population in Uganda. Among participants in South Africa, education, physical health, and perception of the clinic as “too far” were significantly associated with early gaps in ART. These findings can help inform future interventions that promote adherence to ART for this population.
